# Selections and Abstracts

**Published:** 1916-01

**Authors:** 


### SELECTIONS AND ABSTRACTS

### PRINCIPAL CAUSES OF DEATH.

### Census Bureau's Summary of the Statistics for the Registration Area in 1914.

Washington, D. C.—According to a preliminary announcement with reference to mortality in 1914, issued by Director Sam L. Rogers, of the Bureau of the Census, Department of Commerce, and compiled by Mr. Richard C. Lappin, chief statistician for vital statistics, more than 30 per cent. of the 898,059 deaths reported for that year in the "registration area," which contained about two-thirds of the population of the entire United States, were due to three causes—heart diseases, tuberculosis, and pneumonia—and more than 60 per cent. to eleven causes—the three just named, together with Bright's disease and nephritis, cancer, diarrhea and enteritis, apoplexy, arterial diseases, diphtheria, diabetes, and typhoid fever.

The deaths from heart diseases (organic diseases of the heart and endocarditis) in the registration area of 1914 numbered 99,534, or 150.8 per 100,000 population. The death or mortality rate from this cause shows a marked increase as compared with 1900 when it was only 123.1 per 100,000.

Tuberculosis in its various forms claimed 96,903 victims in 1914, of which number 84,366 died from tuberculosis of the lungs (including acute miliary tuberculosis). As a result of a more general understanding of the laws of health, the importance of fresh air, etc., due in part, no doubt, to the efforts of the various societies for the prevention of tuberculosis, there has been a most marked and gratifying decrease during recent years in the mortality from the scourge of civilization. In only a decade—from 1904 to 1914—the death rate from tuberculosis in all its forms fell from 200.7 to 148.8 per 100,000, the decline being continuous from year to year. This is a drop of more than 25 per cent. Prior to 1904 the rate had fluctuated, starting at 201.9 in 1900. Even yet, however, tuberculosis has the gruesome distinction of causing more deaths annually than any other form of bodily illness except heart diseases, and over 40 per cent. more than all external causes—accidents, homicides, and suicides combined.

Pneumonia (including bronchopneumonia) was responsible for 83,804 deaths in the registration area in 1914, or 127 per 100,000—the lowest rate on record. The mortality rate from this disease, like that from tuberculosis, has shown a marked decline since 1900, when it was 180.5 per 100,000. Its fluctuations from year to year, however, have been pronounced, whereas, the decline in the rate for tuberculosis has been nearly continuous.

The only remaining death rate higher than 100 per 100,000 in 1914 was that for Bright's disease and acute nephritis, 102.4. The total number of deaths due to these maladies in 1914 was 67,545, more than nine-tenths of which were caused by Bright's disease and the remainder by acute nephritis. The mortality from these two causes increased from 89 per 100,000 in 1900 to 103.4 in 1905, since which year it has fluctuated somewhat.

Next in order of deadliness came cancer and other malignant tumors, which filled 52,420 graves in 1914. Of these deaths, 18,889, or almost 38 per cent, resulted from cancers of the stomach and liver. The death rate from cancer has risen from 63 per 100,000 in 1900 to 79.4 in 1914. The increase has been almost continuous, there having been but two years—1906 and 1911—which showed a decline as compared with the years immediately preceding. It is possible that at least a part of this indicated increase is due to more accurate diagnoses and greater care on the part of physicians in making reports to registration officials.

Diarrhoea and enteritis caused 52,407 deaths in 1914, or 79.4 per 100,000. This rate shows a marked falling off as compared with the rate for the preceding year, 90.2, and a very pronounced decline as compared with that for 1900, which was 133.2. Nearly five-tenths of the total number of deaths charged to these causes in 1914 were of infants under 2 years of age.

Apoplexy was the cause of 51,272 deaths, or 77.7 per 100,-000. The rate from this malady has increased gradually, with occasional slight declines, since 1900, when it stood at 67.5.

Arterial diseases of various kinds—atheroma, aneurism, etc.—caused 15,044 deaths, or 22.8 per 100,000, in the registration area.

No epidemic disease produced a death rate as high as 18 per 100,000 in 1914. The fatal cases of diphtheria and croup—which are classed together in the statistics, but practically all of which are of diphtheria—numbered 11,786, or 17.9 per 100,000, in that year, the rate having fallen from 43.3 in 1900. This decline of nearly 59 per cent. is relatively greater than that shown by any other important cause of death. The rate has not fallen continuously, but has fluctuated somewhat from year to year.

Diabetes was the cause of 10,666 deaths, or 16.2 per 100,000. The rate from this disease has risen almost continuously from year to year since 1900, when it was 9.7 per 100,000.

The mortality rate from typhoid fever has shown a most gratifying decline since 1900, having decreased from 35.9 per 100,000 in that year to 15.4 in 1914, or by 57 per cent. This decline has been almost as great, relatively, as that for diphtheria, and has been greater than that for any other principal cause of death. The total number of deaths due to typhoid fever in 1914 was 10,185. The marked decrease in the mortality from this disease given emphatic testimony to the effectiveness of present-day methods, not only of cure but of prevention. The efficacy of improved water-supply and sewerage systems, of the campaign against the fly, and of other sanitary precautions is strikingly shown by the reduction of the typhoid mortality rate to the extent of more than five-ninths in 14 years.

WHOOPING COUGH, MEASLES, AND SCARLET FEVER.

The principal epidemic maladies of childhood—whooping cough, measles, and scarlet fever—were together responsible for no fewer than 15,617 deaths of both adults and children, or 23.7 per 100,000, in the registration area in 1914, the rates for the three diseases separately being 10.3, 6.8, and 6.6, respectively.

In 1913 measles caused a greater mortality than either of the other diseases, but in 1914 whooping cough had first place. In every year since and including 1910, as well as in several preceding years, measles has caused a greater number of deaths than the much more dreaded scarlet fever. The mortality rates for all three of these diseases fluctuate greatly from year to year. The rates for measles and scarlet fever in 1914 were the lowest in 15 years, while that for whooping cough was considerably above the lowest recorded rate for this disease, 6.5 in 1904, although far below the highest, 15.8 in 1903.

### Railway and Street Car Accidents.

Deaths due to railway accidents and injuries totaled 7,062, or 10.7 per 100,000. This number includes fatalities resulting from collisions between railway trains and vehicles at grade crossings. The death rate from railway accidents and injuries is the lowest on record and shows a most marked and gratifying decline as compared with the rate for 1913, which was 13 per 100,000, and a still more pronounced drop from the average for the five-year period 1906-1910, which was 15 per 100,000.

Deaths resulting from street car accidents and injuries numbered 1,673, or 2.5 per 100,000. This rate, like that for railway facilities, is the lowest on record and shows a material falling off as compared with 1913, when it was 3.2, and as compared with the average for the five-year period 1906-1910, which was 3.7.

### Suicides.

The number of suicides reported in 1914 was 10,933, or 16.6 per 100,000 population. Of this number, 3,286 accomplished self-destruction by the use of firearms, 3,000 by poison, 1,552 by hanging or strangulation, 1,419 by asphyxia, 658 by the use of knives or other cutting or piercing instruments, 619 by drowning, 225 by jumping from high places, 89 by crushing, and 85 by other methods.

### DERMATITIS CAUSED BY PRIMULA POISONING.*

BY DOUGLASS W. MONTGOMERY, M. D., and GEORGE D. CULVER, M. D.

Primula poisoning is probably not frequent, but curiously enough in the past few months we have run across quite a number of instances. Its previous infrequency may have been either due to absolute infrequency or to oversight on our part. In medicine one usually finds only what one looks for, and in primula poisoning, as we shall see later, the cause must frequently be persistently searched out or it will not be found. The following case illustrates the intensity the disease may assume, and its elusive etiology:

A woman, forty-four years of age, consulted us August 26, 1913, on account of a blotchy, intensely itchy erythema of the face, and a papulo-erythematous eruption of the flexor surface of the forearms, that had begun five months previously. Inquiry was made in regard to both rhus and primula poisoning, but the possibility of either was denied. The long, steady endurance of the eruption would exclude rhus poisoning, as the exposures to poison oak, in contradistinction to primula exposures, are interrupted.

These external causes of dermatitis being excluded, internal incitants to vaso-motor disturbance were sought for, and enough was found to account for the trouble. She had symptoms of slight icterus with a somewhat enlarged liver, and an immensely dilated colon, with tenderness in the left inguinal region. The urine contained indican, and showed also a green yellow line with the cold $H N O^3$ test, that we assumed to be due to bile. She was taking about a quart of buttermilk a day, which represented a decided intake of cheese, and she was eating a considerable quantity of fruit, the fructose of which is often very irritating to the skin.

She frequently had to take a soporific to induce sleep, and she had pains across the lower part of the abdomen, which she said she had often noticed preceded the outbreak of an eruption.

The most varied things seem to give rise to the eruption, as, for instance, a drop in the atmospheric pressure preceding a rain storm, or sitting up late at night to play cards, or the mental irritation caused by a wearisome relative. On January 23, 1914, an earthquake seemed to bring out two red spots, one on each cheek. Everything indicated the existence of an exceedingly labile vasomotor system, especially of the face and upper extremities, situations in which such vaso-motor eruptions tend to appear.

The first effect of our treatment also confirmed us in our view that we were correct in considering the disease a vaso-motor neurosis, as we were apparently strikingly successful, and were congratulated on our good fortune by a physician who previously had treated the patient.

During the summer she went to the country where she became perfetcly well, through bad weather, card parties, and all other causes that seemed to be so deleterious while living in the city. Her husband wrote to us that he especially tried her out in these directions. Finally, she arrived home on the morning of July 28, and by the evening she was well on her way for another bad attack. She fled to the country again, and recovered speedily and completely. Immediately on hearing of this we wrote to the husband to get an expert gardener, and to search the house and grounds for primroses. They were easily found; they were around a fountain, and were the picture of innocence and loveliness. The patient did not know primroses by their name. The gardener subsequently admitted that he knew primroses caused trouble on the skin. Naturally, however, he was more interested in selling them, than in telling of their disagreeable effects.

On thinking back, the vagaries of the eruption were accounted for. For instance, the patient had helped her husband during the rush of the Christmas trade, and as a consequence was not so much at home, and had a marked respite, that we all ascribed to the treatment.

The erythema in this case was an intense dusky redness, especially marked across the middle zone of the face, with puffiness of the lower lids. The redness had the peculiar bronze tint seen in chrysarobin dermatitis, and gave the patient an Indian-like complexion, and after its subsidence a brown pigmentation remained. Once the eruption appeared as an itchy erythema of the privates. An attack was often accompanied by the appearance of vesicles and blebs. The itchiness and burning were so severe as often to require strong sedatives, both internally and topically.

In investigating the etiology of such an effection the physician necessarily must leave much to the patient. For instance, in this case, we remember distinctly that we early asked about the possibility of primroses as a cause, and being assured on this point, the idea of the affection arising from internal disturbances was taken up. In another case that one of us saw with a friend, the same lapse occurred; the inquiry regarding primroses was made, with a negative response, and it was only after an absence from home with recovery, and a return home with a relapse, that a more thorough search revealed the cause.

Very shortly ago we were consulted by a young man with a light red, mottled erythema of the face and hands, and an urticarial eruption of the body. An inquiry about primroses elicited a negative response. At the next visit a more persistent questioning induced the patient to look into his garden more closely. He found the criminals; they had escaped observation because of not being in bloom, and because the leaves are low and inconspicuous. In this case the eruption was most marked on the left side of the face, the side on which he wore his button-hole flower.

It is quite characteristic of the affection that it should disappear on the patient leaving home, to reappear on returning. Foerster had a patient that got an attack on Easter Sunday afternoon for three successive years. Each time the "gouty eczema" was cured after a stay at a German health resort.

The face, hands and arms, according to Foerster, are the almost exclusive situations of the dermatitis. We have seen it involve the trunk as an urticaria-like eruption, and, like poison oak, it may appear on the privates, although this is rare. As Foerster has remarked it may show itself only as "an intensely itching or burning sensation confined to the finger pulps, which become puffed, tense and slightly reddened."

H. A. Sharpe has drawn attention to the occurrence of this form of eczema in the country from the wild primrose. He thinks that it often occurs from handling the udders of cows that have walked among the dew-laden flowers.

In making a diagnosis it is often a pitting of our wits against the traps laid for us by Nature, and frequently it is Nature that wins out, and she may do so when we feel most confident, as for instance, for a long time in the first case here recited. Conditions in the alimentary canal are so often the key to eruptions on the skin and mucous membranes, that they constitute a temptation not to look for topical causes. When, however, in such a case the cause happens to be found by an outsider what a deal of opprobrium and ridicule the physician is subjected to! And we freely admit that in this case it was not an intellectually worked out diagnosis, but a chance thought that finally solved the problem.

PRIMROSE—PRIMULA SINENSIS

One of the finest spring blooming pot plants. Easy to grow, pretty in leaf, handsome in flower, and continuously in bloom for months at a time. These merits alone would earn a place in every window; but when we add that it is one of the plants never attacked by insects and that it will bloom in a sunless window —where a Geranium or Heliotrope would never show a bud—it is clear that the smallest collection would never be complete without it. Our seed is saved from the choicest strains and can be relied upon to produce the largest flowers and finest colors. —Mixed—Large packet 25c. Plant from February to May.

The advertised description of the plant is included because it shows so well the attitude of the florist. This desire on the part of the flower dealer to sell the plant, together with the beauty of the flower and its good blooming qualities that appeal so strongly to women and induce them to buy, will, we have no doubt, successfully prevent the elimination of this interesting dermatitis.

It is also interesting to note that the plant is seldom attacked by inserts. Probably the same poison that causes the dermatitis in human beings also acts poisonously on insects, and so constitutes a defense for the life of the plant.

---

### DISEASES OF THE EPIDIDYMIS AND TESTICLE.

By Henry H. Morton, M. D., Brooklyn, N. Y.

Epididymitis is more frequently seen than orchitis. Causes are gonorrhea, traumatism, passage of urethral instruments, or as a sequel to prostatectomy. The organisms reach the epididymis through the vas deferens by means of its peristaltic action which moves in either direction.

Causes of orchitis are syphilis, malignant disease, or metastasis during an attack of mumps.

Gonorrheal Epididymitis: Testicle red, swollen, tender, inflamed. The epididymis is enormously swollen and surrounds the testicle. A small quantity of hydrocele fluid is usually found. Discharge from urethra usually ceases while epididymis is affected and begins again as the inflammation subsides.

Treatment: Preventive: Have patient wear suspensory and keep as quiet as possible. No urethral instruments should be passed and no forced injections given.

When present the patient should be put to bed, the scrotum supported with a handkerchief bandage, and continuous hot applications applied. Hot flaxseed poultices, or hot lead and opium work very well.

Cold is no longer used as it is liable to leave a hard tough infiltration of the epididymis, thereby causing sterility.

In moderately severe cases accompanied with great pain 20 per cent. guaiacol ointment covered with cotton and heat usually allays the suffering.

In recurring epididymitis or in very severe cases which do not respond to treatment the Hagner operation is indicated.

Where there is a great deal of hydrocele fluid present, aspiration of the fluid makes the patient comfortable.

Where the epididymis has been blocked as the result of inflammation and the patient sterile, the patent end of the epididymis may be transplanted into the testicle and a new channel for the exit of the spermatoza is made. In about 50 per cent. of cases operated the sterility is relieved.

Tuberculosis of the Epididymis: Begins as a small painless lump which gradually increases in size.

The epididymis is usually infected:

1. By hematogenous infection.
2. By extension from higher up; vesicles, prostate, kidneys.

Later the testicle becomes infected, and breaks down and becomes filled with pus.

The focus may remain latent for a long time, and then be lighted up by some traumatism.

Treatment: Epididymectomy is the operation of choice when the testicle is not involved, but when the testicle is affected Castration is indicated.

After the patient recovers from his operation he should be instructed as lead an out-of-door life as ordered in the case of any other Tuberculous individual.—New York Medical Journal for February 13, 1915.

---

### THE PROSTITUTE: AN ETHNOLOGICAL STUDY.

By Lee Alexander Stone, M. D., Member American Anthropological Association; American Genetic Association; Southern Sociolcgical Congress, Memphis, Tenn.

My reason for writing this article is that for centuries all forms of prostitution have been classified under one head by Christians and reformers. They have refused to believe that female human beings could have been impelled by religious motives to sacrifice their virginity at hymen's altar for the purpose of improving their race.

I hope to make my disquisition so clear that my readers who expect me to deal in pornographic rhetoric will be disappointed. I have no patience with the individual who is constantly trying to find "dirt" in every thing he reads, which deals with the actions of man and his mate, or with the doings of individuals who have been forced to become anti-social creatures by an uncharitable and unfeeling society, who only recognize "the sin of being found out" as being the one which cannot be forgiven. I feel a great sympthy for the present-day prostitute. She comes from the proletariat and as a consequence of this educational workers and religionists have not thought her worth while as a factor in society. They have not tried to educate her kind; by her kind I mean the great mass of ignorant individuals who are in our country to-day, who have knocked many times at the doors of high-brow institutions and ponderous religious systems, begging to be allowed entrance into the holy of holies of a greater enlightenment, only to be turned away by those who should have thrown the mantle of charity about their shoulders and taken them in. All Americans are snobs, the dollar is the only thing that counts, and even though I may be contradicted by individuals who will say that philanthropists abound who are trying to lift those who cannot help themselves out of the depths,—their work so far has amounted to little. They have put the cart before the horse, they have failed to see the underlying reasons for the existence of a pauper class who are totally unable to help themselves, and who need real help, help not alone of a financial nature, but of an educational type that will pull them out of the mire of despair and give them sufficient mental strength to resist the temptations that beset them in so many forms.

Altruism is a bugbear in modern society and men and women who feel a real desire to help are called "dreamers" or "agitators," and are crucified on the cross of materialism. The individual who dares to express his or her opinion unless it agrees with that of the ultra-conservative or, better expressed, the provincial member of society, is damned. He or she may finally come to the top, but more often are they crushed by the Juggernaut of ridicule until they give up in disgust, feeling that it is useless to longer tell the truth to ears that are deaf.

If individuals are to progress and society is to develop to heights unattainable through ignorance, the truth must be told. Nothing must be regarded as sacred unless it will stand in the strong light of fact, and bias should have no place in the minds of educators or investigators. We are too prone to jump to a conclusion and allow that conclusion to govern our action. We should not, until after a careful investigation has been made, announce our opinions for fear of doing damage where it was not intended that damage should be done. The truth makes men and women whole and replaces readily doubt and hypocrisy.

Many of my readers will be shocked when I say that the primitive prostitute was of the greatest benefit to society.

People of ages and ages ago were endogamous, they absolutely forbade the marriage of one of their clan or gens to a member of an outside-organization or gens. As a result of this it was not long before all the members of a single clan or gens were related by blood ties to one another and the natural consequence of consanguinous union—physical and mental degeneration—resulted. Many females violated the rules of their clan and had intercourse with male members of gens who did not belong to their own, and gave birth to offsprings who became stronger both physically and mentally than those born as the result of consanguinous union. The wise men of the tribe were the first to notice this improvement as the result of promiscuous intercourse with outsiders and consulted the priests for advice. Religious prostitution was the result. Rather than become exogamous all single females were required once in a life-time to submit themselves to a stranger. According to our present code of ethics we associate a procedure of this sort with the sensual and immoral. The sacrifice of virtue to the goddess Mylitta was not in any sense a sensual or lewd custom. It marked a desire to progress, and its application as a religious duty was highly proper. The priests realized that unless outside blood was imported into their clans their doom was sealed; that the mark of inbreeding would leave its indelible stain upon them and that degeneracy and its evil brood would soon destroy them.

Sacred prostitution permitted females to have children by outsiders without the obligation of marriage; thereby bringing new blood into their tribes which strengthened them without the importation of a foreign influence. Prostitution as an original sacred institution stood foremost as being of service to mankind. Without it the human race would possibly have decayed and progress would have been rendered impossible.

The family as it originally stood was composed of a host of females with but one male, who attended to all of their sexual needs. The patriarch, as he was called, jealously guarded all of his women even to the extent of driving his sons from his camp as soon as they reached puberty and forbade them entering it ever again under the penalty of death. He had children by his wife, his sisters, his daughters and his daughter's daughters. This condition existed for centuries until an element of spirituality entered the heart of a mother who persuaded the patriarch to allow her favorite son to remain with the injunction that he was to let his father's females alone. He was permitted to capture a wife and bring her home and establish a clan of his own. This finally resulted in the establishment of settlements with one common ancestor and all bore his name.

You can readily see what disastrous results might be expected when a father had intercourse not only with his wife and her sisters, but with his own daughters. Weaklings were born and something had to be done to strengthen, hence females became the logical savious of their race.

It was centuries after religious prostitution came into existence that an exogamous union was permitted. When exogamy became an established fact, and men and women were forbidden to marry within their own clans, human kind began to progress as it never had done before. Tribes became civilized, a desire to push ahead of neighboring clans obsessed them as a result of this great nations were born. Babylon, Phoenicia, Egypt, Greece and Rome with their ascending greatness came into existence to leave their stamp of civilizing influence to be felt for centuries upon centuries after their greatness had become merely a matter of historical record.

Necessity has been the cause of progress. Through their need for better blood and a stronger people, the ancients conceived the sacrifice of their females to the Gods of procreation in order that new blood might become fused with their's for their physical, psychical and mental betterment. Their minds were primitive, therefore they could not conceive of schemes our enlightened minds would devise in a moment for their betterment, without the sacrifice of the virginity of their females. They had no conception of marriage in the way that it is today understood. They did their best. The procreation of the species was their aim in life and the infusion of new blood which would strengthen not only them but clans to come, was the desire closest to their hearts.

It is thought by some that sacred prostitution occurred only in temples or gardens dedicated to the goddess Mylitta in Babylon. This is not true, for all worshipers at the many shrines of Venus were only obeying the demands made by the procreative instinct. This same curious custom of religious prostitution existed among the Lydians, Syrians, Carthagimans, Egyptians and Greeks, and to a limited degree among the Romans, who permitted prostitutes to frequent their temples and ply their trade. These same Roman prostitutes rendered a tithe to the temples dedicated to the gods of that period, and in many instances their gifts were magnificent.

The goddess Flora or goddess of the flowers of the Greeks was a noted courtesan who made an enormous fortune, which she left to the state. Her legacy was accepted, and the Senate, in gratitude, decreed that the name of "Flora" should be inscribed in the fasti of the State and that solemn fetes should perpetuate the memory of her generosity. These fetes, connected as they were also with the goddess of fecundity, were accompanied by scenes—that would be accorded scandalous today—in the circus.

Sacred prostitution at Athens was under the patronage of Venus Pandemos, who is said to have been the first divinity that Theseus caused the people to adore, or, at least to whom a statute was erected on the public place.

It was a common practice in Greece to consecrate to Venus a certain number of young girls, when it was desired to render the goddess favorable, or when she had granted the prayers addressed to her.

Gautama was entertained at Vesali by a lady of high rank who had the title "Chief of the Courtesans." Jesus of Nazareth, like Gautama of the Hindoos, was entertained by a most charming woman who had aspired to the title of "Chief of the Courtesans" of Jerusalem. Christ did not feel at all offended at her entertainment and rebuked his disciples for attempting to interfere with Mary Magdalene when she anointed His feet with oil and wiped them with her hair.

Prostitutes before the Christian era enjoyed a position that was unique. They were usually well educated and charming women, women who were capable of making themselves felt by the power of their intellects, as did the Hetaire of Athens, as well as by alluring charms they were capable of displaying.

In Armenia strangers alone were entitled to seek sexual hospitality in the sacred enclosures of the temple of Anaitas; and it was the same in Syria during the fetes of Venus and Adonis. Armenian women who became sacred prostitutes after a period of service in the temples were much sought after as wives, because it was thought that their service rendered them better fit for a normal sexual life as married women than would women who had not been introduced into the mysteries of the sexual embrace. I am told that today in portions of Austria and Armenia women frequently become prostitutes to earn their dower money and that their former profession in a way affects their standing with their prospective husbands.

The Jews, prior to the time of Joshua, the prophet, maintained in their temples priestesses who supplied the sexual needs of male worshipers for a fee that was paid into the temple for its support. The ramifications of sacred prostitution have penetrated the many institutions belonging to religious organizations for thousands of years.

Mankind has followed the demands of sex-hunger the world over. Sex-hunger with its gratification caused the establishment of the deity Phallus, and remains of phallism may be found in every creed and sect extant.

The former custom of offering a guest, a daughter or wife as a sexual partner for a night came from the temple of Mylitta and was, in a way, a modified form of sacred prostitution. It was done as a sacrifice to the goddess of fecundity, aside from a desire to satisfy the sexual needs of the guest and to appear hospitable.

In Japan a girl enters the Yoshiwara for a term in order to propitiate the gods. She sacrifices her virginity as an act of service to her parents. She does not lose caste nor does she find it a difficult matter to obtain a husband.

Unfortunately during the first thousand years after the birth of Christ, women fell very low in the moral scale, due not to themselves but to the damning attitude of the church towards their sex. Priests taught that women were fit only for sexual and sensual gratification and that they were agents of the evil one. It amy be stated, and truthfully, that some women even in the atmosphere of the contempt for their sex in which they lived, succeeded in climbing to enviable positions and became advisors in the councils of many of the higher churchmen.

Priests advocated celibacy for men and women alike and many married only to maintain the strictest continence, never allowing themselves the joys of the marriage bed. They became the brides and bridegrooms of Christ and the church. The history of Sacerdotal celibacy is filled with causes for the social decay of the "dark ages." Men and women hungered for sexual contact but, on account of threatened religious disapproval they abstained. Abstinence from the pleasures of the conjugal relation caused them to become more or less decadent. Ambition ceased to stir their souls, hence they became nothing more than automatons under the guidance and control of priests.

About seven hundred A. D. a reaction set in and the Troubadours of Provence came into their own. They sang of their loves, wrote poetry of a most passionate type to the ladies whose favors they desired to gain, and, as a consequence of this, women again came into what was rightfully their own—the right to love and be loved by males.

Had it not been for the influences, according to Lucka, of the Italian poets Dante and others, woman would have been deified and would have replaced Jehovah as a God. Through their influence Mary became the mediator and was worshiped as such. Artists began to paint madonnas and used as their models their mistresses. It is said by some investigators that hardly a madonna has been painted but that its face is the face of the beloved mistress of the artist.

Prostitution in is sacred form, as practiced by the ancients, did not imply the loss of virginity in the sense in which we understand such a loss today. The prostitute was respected because she was obeying the command of the procreative principle in nature. So throughout the ages prior to the advent of christianity women sought to bring into the world offspring that would reflect credit, not only on them but on the deity worshipped.

It was Solon who, about five hundred years B. C. started our present system of commercialized prostitution. He had quarters built for slave girls who sold their charms to any who came to call on them. Solon believed that he was protecting the home, that he was eliminating any chance for married women to be insulted or to have their virginity disturbed by those seeking sexual pleasures. We see then that prostitution as a modern institution was established to protect the female members of the home from the advances of sensualists, and that it differs in every respect from the ancient observance of sacred prostitution, which was for the injection of new blood into endogamous clans and to propitiate the gods of procreation.

The first form of prostitution, we can readily see, was more or less of a truly religious type, whilst the second form represents the highest type of sensuality. It shows that man catered to his appetites to such a degree that the debasing of women had to be stopped by placing on sale the bodies of women who were very low in the moral scale and who in their original position in society were slaves. The present-day prostitute like her ancient relative who gained her position, that of being a social outcast by the command of Solon, has not elevated herself in the social scale, but has fallen lower and lower until now she is not alone a sensualist but a carrier of disease as well, with its degenerating influence on the offspring of her associate who frequently leaves her side diseased to marry a virgin, only to infect her and cause her to suffer untold agony and, when conception occurs, to carry a disease to her offspring that will affect it physically and mentally even to producing idiocy, bone discrasias, mental and moral degeneracy.

After studying some of the internal conditions that Europe is facing at the present moment, it is my honest belief that a rebuilding of our moral code will occur after the war is over. The tremendous loss of life among the male population has already caused sociologists to wonder how the birth-rate will be kept up. One of two things is bound to happen; first a polygamous union between one strong male and a number of females may be advocated by those seeking to rehabilitate the state and bring its population up to standard; second, forms of prostitution may be tolerated in order that women may have the opportunity to satisfy their maternal instincts and bear children, thereby filling in the ranks of those who have lost their lives as a result of war. The governments wherein these children are born will legitimatize them and look after their welfare.

It will be found absolutely necessary to establish new standards of morals if Europe is to come into her own again. The blending of Occidental with Oriental opinion that is going on among the nations at war because of Oriental troops fighting alongside of Occidental soldiers for one common cause, is bound to have its effect and men's intellects will be broader than ever before after the war is over. They will be more tolerant and will refuse to live longer under the guidance of a system of morals that has kept hypocrisy and its brood alive for centuries. A religion of tolerance will be the outcome of this blending of ideas and no longer will Christians be able to hurl anathemas of orthodoxy at the heads of so-called heathens. They will no longer be charged with worshiping false gods and idols of paganism. Our refrence to humans as pagans is only to display our ignorance, and to confess that because they differ with us in their views of a deity we have become angered at them, that we are bigoted to the extent of accrediting to ourselves rights that are not even assumed by gods. Heathens will cease to exist and under a united brotherhood will all men seek enlightenment for the elevation of the race.

The wonderful Oriental teachings of Christ and Gautama or Buddha will be better understood and for the betterment of mankind, and the elevating ideals for which they stood will redound to the credit of all races extant, and a broader and more enlightening civilization will come that will destroy all that is false and bring truth into its own.

The mantle of charity will fall on the shoulders of mankind, and men and women will view with a broader scope of vision institutions which in the past have been looked upon as being immoral, and will attempt to eliminate them by educating individuals to see the folly of their existence.

The days of religious fanaticism are gone, no longer will men be able through an esoteric mysticism to control. Modern as well as ancient inquisition days are over and shortly will man throw aside the cloak of orthodoxy and sectarianism that has hindered him for centuries, to don the robes of the teacher of an altruistic philosophy which will develop humanity's spiritual side as it never has been developed before. The search light of truth is shining on dark corners in society and routing out false teachers of a falser code of ethics. Shortly all that will remain of the dark ages of twenty centuries will be a memory that will be hidden in the archives of history only to be viewed in centuries to come as being fictional. Races which have not yet made their appearance and will not for generations, will hardly believe that mankind could have ever lived under the control and guidance of false philosophies, which almost crushed them out of existence and made them intolerant and narrow to the extent of inventing horrible punishments for unbelievers.

I have no doubt but that I will be charged with being a dreamer who is trying to hasten the day when an Utopia will exist and man will no longer have cause to worry—that day will never come. Our present worn out religious asceticism with its hypocrisy and chicanery will die, and the gods of yesterday and today will be relegated to the dead lumber room of the past in order to make a place for spirituality and a true religion, a religion that can offer a promise of salvation to all alike, which can be developed through individual effort.

The religion of the future will be a collective something and will be referred to by worshipers as "our religion," instead of as it is referred to today as "my religion." The selfish sect and creed will be totally eliminated. The above tolerance and freedom will be blood-bought by those soldiers in Europe who are sacrificing their lives in order that enlightenment of the right sort may come to those who are left. Therefore, we may truthfully say that the great war will accomplish good in the future. —The Urologic and Cutaneous Review, November.

1190 Linden Avenue.

